# Polyomavirus-Associated Trichodysplasia Spinulosa Involves Hyperproliferation, pRB Phosphorylation and Upregulation of p16 and p21

**DOI:** 10.1371/journal.pone.0108947

**Published:** 2014-10-07

**Authors:** Siamaque Kazem, Els van der Meijden, Richard C. Wang, Arlene S. Rosenberg, Elena Pope, Taylor Benoit, Philip Fleckman, Mariet C. W. Feltkamp

**Affiliations:** 1 Department of Medical Microbiology, Leiden University Medical Center, Leiden, South-Holland, The Netherlands; 2 Department of Dermatology, UT Southwestern Medical Center, Dallas, Texas, United States of America; 3 Department of Dermatology, MetroHealth Medical Center, Case Western Reserve University, Cleveland, Ohio, United States of America; 4 Department of Pediatrics, University of Toronto, Toronto, Ontario, Canada; 5 Department of Dermatology, Medical University of South Carolina, Charleston, South Carolina, United States of America; 6 Division of Dermatology, University of Washington, Seattle, Washington, United States of America; Virginia Commonwealth University, United States of America

## Abstract

Trichodysplasia spinulosa (TS) is a proliferative skin disease observed in severely immunocompromized patients. It is characterized by papule and trichohyalin-rich spicule formation, epidermal acanthosis and distention of dysmorphic hair follicles overpopulated by inner root sheath cells (IRS). TS probably results from active infection with the TS-associated polyomavirus (TSPyV), as indicated by high viral-load, virus protein expression and particle formation. The underlying pathogenic mechanism imposed by TSPyV infection has not been solved yet. By analogy with other polyomaviruses, such as the Merkel cell polyomavirus associated with Merkel cell carcinoma, we hypothesized that TSPyV T-antigen promotes proliferation of infected IRS cells. Therefore, we analyzed TS biopsy sections for markers of cell proliferation (Ki-67) and cell cycle regulation (p16^i*nk4a*^, p21*^waf^*, pRB, phosphorylated pRB), and the putatively transforming TSPyV early large tumor (LT) antigen. Intense Ki-67 staining was detected especially in the margins of TS hair follicles, which colocalized with TSPyV LT-antigen detection. In this area, staining was also noted for pRB and particularly phosphorylated pRB, as well as p16*^ink4a^* and p21*^waf^*. Healthy control hair follicles did not or hardly stained for these markers. Trichohyalin was particularly detected in the center of TS follicles that stained negative for Ki-67 and TSPyV LT-antigen. In summary, we provide evidence for clustering of TSPyV LT-antigen-expressing and proliferating cells in the follicle margins that overproduce negative cell cycle regulatory proteins. These data are compatible with a scenario of TSPyV T-antigen-mediated cell cycle progression, potentially creating a pool of proliferating cells that enable viral DNA replication and drive papule and spicule formation.

## Introduction

Trichodysplasia spinulosa (TS) is a follicular skin disease observed only in severely immunocompromized patients [1], [2]. The disease is characterized by papules and keratotic trichohyalin-rich spicule formation that involves body extremities and the trunk, but most noticeable the face. Furthermore, thickening of the skin is seen, sometimes accompanied by alopecia of eyebrow hairs and eyelashes [3]. Histological analyses of TS lesional skin biopsies showed acanthosis of the epidermis and disproportionate enlargement of the hair follicles [1], [3]. Compared to normal hair follicles, TS hair follicles seem devoid of normal hair shafts and papilla. Instead, they include large numbers of eosinophilic, trichohyalin-positive cells, probably inner root sheath (IRS) cells, and corneocytes that fill the infundibula of the follicles [4]. In the initial case report by Haycox and colleagues in 1999, excessive Ki-67-staining was observed indicative of follicular cell proliferation [5].

In 2010, from plucked TS-spicules we identified a virus that was called the trichodysplasia spinulosa-associated polyomavirus (TSPyV) [6], [7]. Several later reports have confirmed the presence of TSPyV in TS lesions suggesting its involvement in TS pathogenesis [8]. The presence and high load of viral DNA exclusively in TS lesions, and TSPyV VP1 capsid protein expression restricted to the affected follicles, indicate a causal relationship between active TSPyV infection and manifest TS disease [1]. The pathogenic mechanism employed by TSPyV is unknown so far, but may involve induction of proliferation of TSPyV-infected IRS cells to accommodate TSPyV DNA replication.

It is well known that other, better-studied polyomaviruses such as SV40 and Merkel cell polyomavirus (MCPyV) encode proteins that counteract host regulatory cellular factors and induce cellular transformation [7], [9]. The polyomavirus large tumor (LT) antigen is generally considered the most potent viral transforming protein that revokes many functions of cellular factors, to the benefit of the virus life cycle [10], [11]. One of its important functions is to induce cell cycle progression by inactivation of the retinoblastoma protein family members (e.g., pRB) [12]. Through its conserved LXCXE motif, LT-antigen interacts with pRB tumor suppressor protein and deprives it from its cell cycle inhibitory function by inducing pRB hyperphosphorylation [13], [14].

This mechanism of cell cycle deregulation is not exclusive to polyomaviruses, as other DNA viruses like human papillomavirus 16 (HPV16) exploit similar regulatory function through the viral oncogenes E6 and E7 [15], [16]. HPV16-positive cervical cancers are highly proliferative as a result of pRB cell cycle control inhibition that consequently leads to p16*^ink4a^* and p21*^waf^* overexpression, and Cyclin-D1 downregulation [17]. p16*^ink4a^* and p21*^waf^* are inhibitors of cyclin dependent kinases (CDK), such as CDK4 and CDK6 that promote pRB phosphorylation and G1 to S phase cell cycle transition [18].

Elaborating on a putative interaction between LT-antigen and pRB, in order to explain the proliferative nature of TS, we sought immunohistological evidence of TSPyV LT-antigen-induced hyperproliferation of TS-affected hair follicles. Within a representative set of archived TSPyV DNA-positive TS sections, proliferation, differentiation and cell cycle progression were assessed by analyzing the presence of cell cycle regulation and proliferation markers Ki-67, pRB, p16*^ink4a^* and p21*^waf^*. Staining patterns of these markers were correlated with detection of TSPyV LT-antigen and trichohyalin locally, as markers for viral infection and TS disease. The observed staining patterns that indicate disruption of the follicular cell cycle pathway are discussed with regard to the underlying disease mechanism, possibly involving TSPyV LT-antigen, and with regard to histological and clinical symptoms of TS.

## Materials and Methods

### Patients and materials

A set of six formalin-fixed paraffin-embedded (FFPE) TS lesional skin biopsies was retrieved as described previously (**Table 1**) [1]. The FFPE sections of healthy skin biopsies from three healthy donors were used as negative (normal) staining controls. These skin samples were collected after informed written consent and handled according to the declaration of Helsinki principles [19]. As a positive staining control for assessment of cellular proliferation and transformation, sections of previously generated human papillomavirus 16 (HPV16) E6/E7 organotypic raft cultures were used [20]. In brief, these organotypic raft cultures were produced using a dermal-like 3T3-fibroblast-containing collagen-gel matrix that was seeded with primary human keratinocytes (PHK) stably expressing HPV16 E6/E7 proteins from the plasmid pLZRS [21], [22]. After 10 days in culture, the organotypic raft cultures were fixed with paraformaldehyde, processed for embedding in paraffin and sectioned afterwards.

**Table 1:** List of analyzed TS samples.

Case ID ^Ref^	Age	Sex	History	Collected	Country	TSPyV load *
**TS4** ^[1]^	**5**	**Male**	**Kidney Tx**	**2009**	**USA**	**1.2E+05**
**TS5** ^[1]^	**63**	**Female**	**Heart Tx**	**2010**	**USA**	**4.4E+06**
**TS8** ^[4]^	**5**	**Female**	**Heart Tx**	**2007**	**Canada**	**5.1E+04**
**TS10** ^[3]^	**5**	**Male**	**Heart Tx**	**2008**	**USA**	**5.6E+05**
**TS11** ^[5]^	**43**	**Male**	**Kidney Tx**	**1997**	**USA**	**1.7E+06**
**TS13** **	**43**	**Female**	**Kidney Tx**	**2012**	**USA**	**2.1E+04**

*, Viral copies per cell measured as described (all) and reported (TS4-TS11) by Kazem *et al.* [1].

**, TS13 concerned a kidney transplantation patient immunosuppressed with tacrolimus, mycophenolate, and prednisone. TS was diagnosed 10 months after the rash was noted. Symptoms improved after reduction of immunosuppression and remained absent ever since.

### Histology and immunofluorescence analysis

Four µm paraffin sections were cut for histological and marker-specific immunofluorescence (IFA) analyses. The sections were heated overnight at 60°C on glass slides and the next day deparaffinized in xylene and rehydrated through descending grades of ethanol to distilled water. Slides for histological assessment were directly stained with Hematoxylin and Eosin (H&E). Sections for IFA analysis were subjected to antigen retrieval in citrate-buffer. After blocking, the sections were incubated overnight with the primary antibodies, listed in **Table 2**, in a moist chamber at 4°C. The day after, the slides were incubated with secondary antibodies at dilutions indicated in the table, and supplemented with Hoechst for DNA staining. Slides were kept in dark and analyzed under a fluorescence microscope and representative pictures were taken with Axiovision software (Carl ZEISS Vision, USA).

**Table 2:** Primary and secondary antibodies used for immunofluorescence.

Primary antibodies (origin)	Clone	Dilutions	Company
**TSPyV LT-antigen (rabbit)** *	V5264	1∶1000	GenScript, USA
**Pre-immune (rabbit)**	V5264	1∶1000	GenScript, USA
**TSPyV VP1-antigen (rabbit)** **	V581	1∶1000	GenScript, USA
**Pre-immune (rabbit)**	V581	1∶1000	GenScript, USA
**Trichohyalin (mouse)**	AE15	1∶250	Santa Cruz, USA
**Trichohyalin (rabbit)**	TCHH	1∶500	Sigma-Aldrich, USA
**Ki-67 (mouse)**	MIB-1	1∶250	Abcam, USA
**p21** ***^waf^*** ** (mouse)**	6B6	1∶250	BD Biosciences, USA
**pRB (mouse)**	G3-245	1∶250	BD Biosciences, USA
**Phospho-pRB (Ser807-811) (rabbit)**	D20B12	1∶250	Cell Signaling Tech., USA
**p16** ***^ink4a^*** ** (mouse)**	JC8	1∶250	Santa Cruz Biotech, USA

Secondary antibodies (origin)	Clone	Dilutions	Company
**Anti-mouse Alexa488-labeled (goat)**	A-11001	1∶300	Invitrogen, USA
**Anti-rabbit Alexa488-labeled (goat)**	A-11008	1∶300	Invitrogen, USA
**Anti-rabbit Cy3-labeled (donkey)**	711-165-152	1∶1000	Jackson, USA

*, Rabbit immunized with two TSPyV LT-antigen-derived synthetic peptides FSSQHDVPTQDGRD (AA, 77–90) and NSRRRRAAPPEDSP (AA, 151–164).

**, Rabbit immunized with TSPyV VP1-antigen-derived synthetic peptide TGNYRTDYSANDKL (AA, 170–183) [1], [29].

## Results

### General histological skin features of trichodysplasia spinulosa

**Figure 1:**
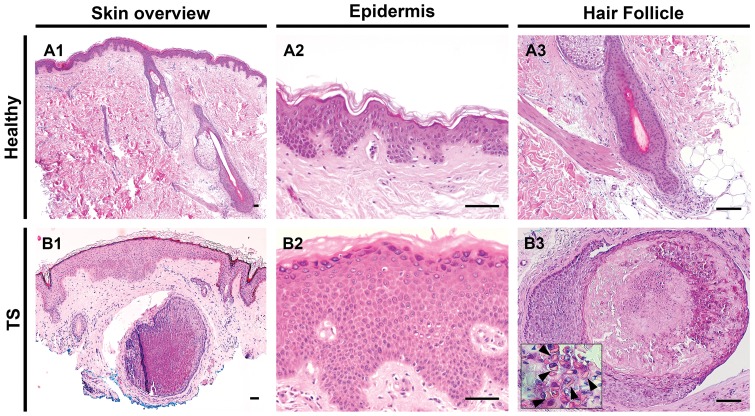
Histological features of trichodysplasia spinulosa. Left column illustrates H&E staining of a low power field of healthy skin (**A1**) and TS lesional skin (**B1**). High power fields of healthy (**A2**) and TS (**B2**) epidermis and hair follicles (**A3** and **B3**). Note the enlarged and dysmorphic hair follicle shown in **B3**, containing eosinophilic granular protein deposits in the cytoplasm of the cells (arrowheads in inset) with abrupt cornification in the center of the follicle. Bars depict 100 µm.

To start, the dermis, epidermis and hair follicles of TS-affected and healthy skin were compared. H&E staining of healthy skin sections demonstrated normal, slim, hair follicles and epidermal stratification (**Figure 1, A1–A3**). In agreement with the literature [1], [3]–[5], in the TS samples enlarged dysmorphic hair follicles were observed (**Figure 1, B1 and B3**). In the center, the TS follicles were inhabited by cells producing eosinophilic protein deposits, possibly trichohyalin protein in the IRS cells. In most TS cases, acanthosis of the epidermis was noted (**Figure 1, B2**).

### Trichohyalin and Ki-67 staining

**Figure 2:**
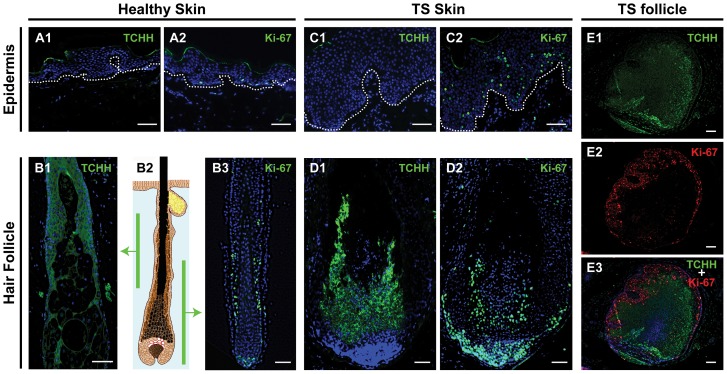
Trichohyalin and Ki-67 expression in healthy and lesional skin. This figure illustrates staining of trichohyalin (TCHH) and Ki-67 in healthy (**A** and **B**) and in TS skin (**C**, **D** and **E**). In the left panel, trichohyalin (**A1**) and Ki-67 (**A2**) staining in healthy epidermis and in healthy hair follicles (**B1** and **B3**) are shown, with the corresponding levels of the follicle illustrated in **B2**. In the middle panel, trichohyalin (TCHH) and Ki-67 staining in TS lesional skin are shown with TS epidermis on top (**C1** and **C2**) and vertical sections of TS hair follicle beneath (**D1** and **D2**). Dotted lines indicate the dermoepidermal junction. Costaining for trichohyalin (green) and Ki-67 (red) of a TS follicle cross-sectioned at the suprabulbar region is shown in the right panel (**E1–E3**). Bars depict 100 µm.

To detect the presence of IRS cells and to pinpoint areas of proliferation in the TS-affected tissue, the sections were stained for trichohyalin and Ki-67, respectively. As expected, in healthy skin trichohyalin staining was detected only along the IRS and absent in the epidermis (**Figure 2, A1 and B1**). Ki-67-staining in healthy skin was restricted to the epidermal basal layer, and to the follicle bulb and the suprabulbar (stem) area (**Figure 2, A2 and B3**). Positive staining of the top cornified layer of the epidermis observed in some of the stained sections in Figures 1 and 2 was considered nonspecific.

In the TS sections, excessive amounts of trichohyalin were observed in the affected follicles, whereas the acanthotic epidermis did not stain for trichohyalin (**Figure 2, C1 and D1**). A substantial increase in Ki-67-positive nuclei was observed both in the follicles and in the TS epidermis, in the latter in basal as well as in suprabasal layers. At the follicle base, a significant increase in Ki-67 expression was evident (**Figure 2, C2 and D2**). Trichohyalin and Ki-67 costaining of a cross-sectioned TS follicle showed trichohyalin staining especially in the follicle center, whereas Ki-67 staining was primarily detected at the follicle margins (**Figure 2, E1–E3**).

### Cell cycle regulation markers and TSPyV LT-antigen expression

**Figure 3:**
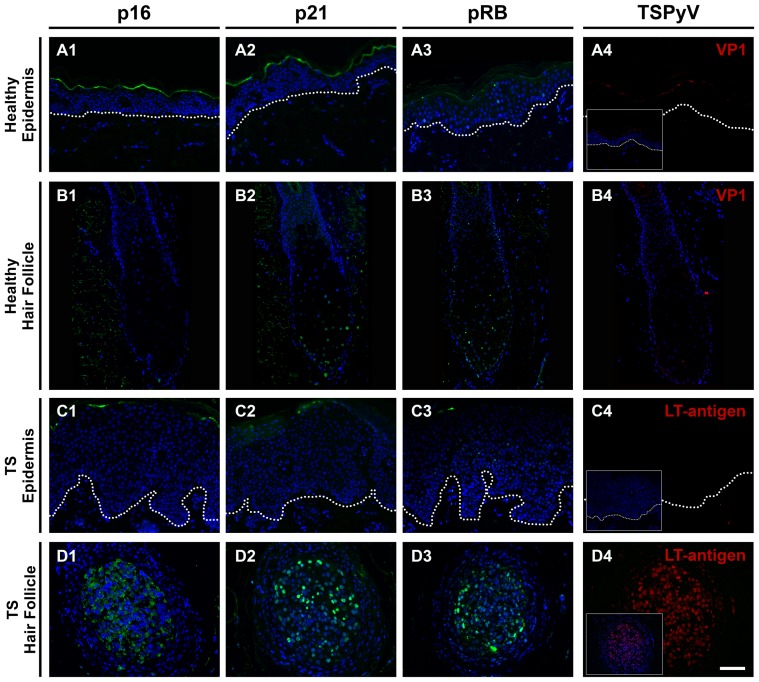
Cell cycle regulation markers and TSPyV LT-antigen expression. Sections of healthy epidermis (**A1–A4**), healthy hair follicle (**B1–B4**), TS epidermis (**C1–C4**) and TS follicle (**D1–D4**) are stained for p16*^ink4a^* (first panel), p21*^waf^* (second panel), pRB (third panel) and TSPyV (fourth panel). Insets in the fourth panel depict the same region with Hoechst DNA staining (blue). Dotted lines indicate the dermoepidermal junction. Bar depicts 100 µm.

To explore the nature of the hyperproliferation in the TS-affected skin, as demonstrated by the increased Ki-67 staining, we investigated locally the expression of major cell cycle regulatory proteins p16*^ink4a^*, p21*^waf^* and pRB. Sections of HPV16 E6/E7-transformed raft cultures were used as positive staining controls (**Figure S1**). Despite occasional faint suprabasal nuclear pRB staining in the TS epidermis, none of these markers were detected in the epidermis of healthy controls or TS cases (**Figure 3, A1–A3 and C1–C3**). A comparable staining pattern was observed in healthy hair follicles, although sometimes faint nuclear staining for p21*^waf^* and pRB was observed (**Figure 3, B1–B3**).

In the TS-affected hair follicles, expression of p16*^ink4a^*, p21*^waf^* and pRB was increased (**Figure 3, D1–D3**). For p16*^ink4a^*, especially cytoplasmic staining was observed and nuclear staining was seen for p21*^waf^* and pRB. These analyses were completed by determining the presence of TSPyV. LT-antigen was detected only in affected hair follicles (**Figure 3, D4**), which also stained positive for p16*^ink4a^*, p21*^waf^* and pRB, suggestive of colocalization of these markers.

### Colocalization of TSPyV LT-antigen, Ki-67 and phosphorylated pRB

Finally, we investigated whether TSPyV LT-antigen expression in the TS sections colocalize with staining of Ki-67 and phosphorylated pRB, in order to explain proliferation induction.

**Figure 4:**
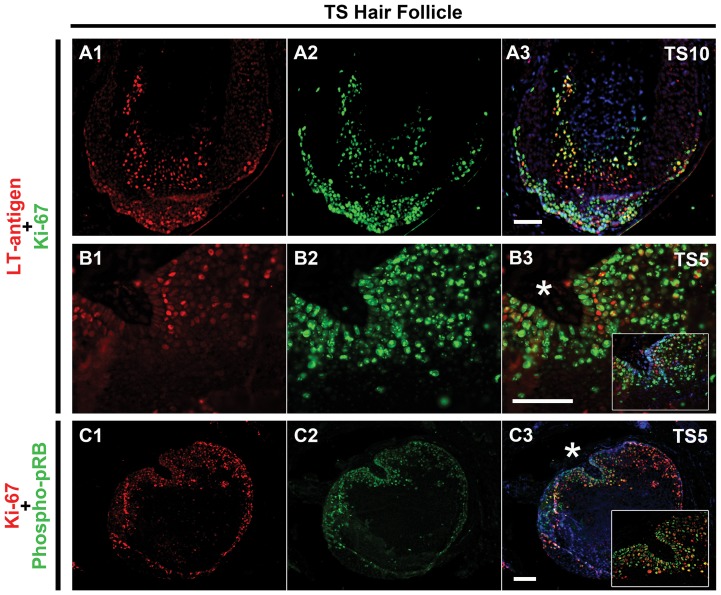
Colocalization of TSPyV LT-antigen, Ki-67 and phosphorylated pRB. TSPyV LT-antigen (red) (**A1**) with Ki-67 (green) (**A2**) and merge (yellow) (**A3**) in a vertical section of hair follicle of TS10 is shown in the upper row. A higher magnification of TSPyV LT-antigen (**B1**) with Ki-67 (green) (**B2**) and merge (yellow) (**B3**) in a suprabulbar cross-sectioned hair follicle region of TS5 is shown in the middle. B3 inset depicts the same region with Hoechst DNA staining (blue). Ki-67 (red) (**C1**) with phosphospecific (Ser807/811) pRB (green) (**C2**) and merge (yellow) (**C3**) in a suprabulbar cross-sectioned hair follicle region of TS5 is shown in the last row. A higher magnification of TS5 margin (**C3, asterisk**) is shown in the inset. Dotted lines indicate the dermoepidermal junction. Bars depict 100 µm.

Double staining for Ki-67 and TSPyV LT-antigen of a vertical-sectioned follicle illustrated that Ki-67-positive cells colocalized with TSPyV LT-antigen expression in the margins of the extended bulbar and suprabulbar region (**Figure 4, A1–A3**). The same colocalization was observed in a suprabulbar cross-sectioned hair follicle (**Figure 4, B1–B3**). When analyzing Ki-67 in combination with phosphospecific pRB, we observed colocalization of Ki-67 and phosphorylated pRB in TS hair follicle margins, indicating hyperphosphorylation of pRB in the proliferating cells (**Figure 4, C1–C3**). A summary of all our histological findings using these markers in individual TS-samples is shown in **Table 3**.

**Table 3:** Overview of cellular and virus markers detected in TS lesions.

	Cellular Markers						Virus Markers	
Case ID	TCHH	p16	p21	pRB	Phospho-pRB	Ki-67	TSPyV VP1 *	TSPyV LT
TS4	+	+	+	+	+	+	+	+
TS5	+	+	+	+	+	+	+	+
TS8	+	NA	+	NA	NA	+	+	+
TS10	+	NA	+	NA	+	+	+	+
TS11	+	NA	+	NA	NA	+	+	+
TS13	ND	NA	+	NA	NA	+	NA	+

*, Reported by Kazem *et al.* [1].

−, negative; +, positive; NA, not available.

## Discussion

In a previous study, in a group of 11 TS patients we established that presence and high load of TSPyV DNA was strongly associated with TS disease [1]. In the same sample-set we showed that viral capsid protein (VP1) expression was exclusively present in distended dysmorphic trichohyalin-positive TS hair follicles [1]. In this first systematic immunohistochemical study of its kind, we analyzed lesional sections from six of these TS patients, which is roughly one fifth of all TS cases reported worldwide [8]. Still, we were unable to perform every staining on all patients, because we were limited in the number of TS sections available for analysis.

Intense Ki-67 staining was detected in the TS-affected hair follicles, especially in the bulbar and suprabulbar marginal regions, indicative of hyperproliferation in these areas. The observed pattern of Ki-67-rich and trichohyalin-poor follicle margins and trichohyalin-rich and Ki-67-poor follicle centers may suggest arrest of proliferation in ‘mature’ IRS cells along central, terminal differentiation of these cells. However, we cannot exclude that different (IRS) [23] cell types explain the difference in margin and center staining.

In addition to increased proliferation of follicular cells, we observed increased Ki-67-staining within the overlying acanthotic epidermis of every TS-patient analyzed. This was observed in particular in the suprabasal layers. To our knowledge, this observation has not been reported previously and possibly implies that TS is not confined to the hair follicles but involves other parts of the skin as well. Whether the observed epidermal hyperproliferation is seen only in the vicinity of a TS lesion, with visible papules and/or spicules, or represents a general feature of TS-patients is not known. In the affected epidermis, we could not detect TSPyV LT-antigen (and VP1-antigen, as analyzed previously [1] (and data not shown)). Therefore, the relationship between epidermal hyperproliferation and acanthosis, and TSPyV infection remains unclear.

Subsequent analyses of the proliferative hair follicles for p16*^ink4a^*, p21*^waf^* and pRB demonstrated a pattern that is known for tissues infected by small DNA viruses involved in cellular transformation, such as SV40 and HPV16 [24]. The staining pattern was indeed exemplified by the staining pattern of the HPV16 E6/E7-expressing organotypic skin cultures included as a positive control, where increased Ki-67 staining was seen together with increased detection of p16*^ink4a^* and p21*^waf^* (Figure S1). This observed association suggests that a comparable disruption of the pRB-dependent cell cycle regulation pathway may be involved in TSPyV infection and TS development.

**Figure 5:**
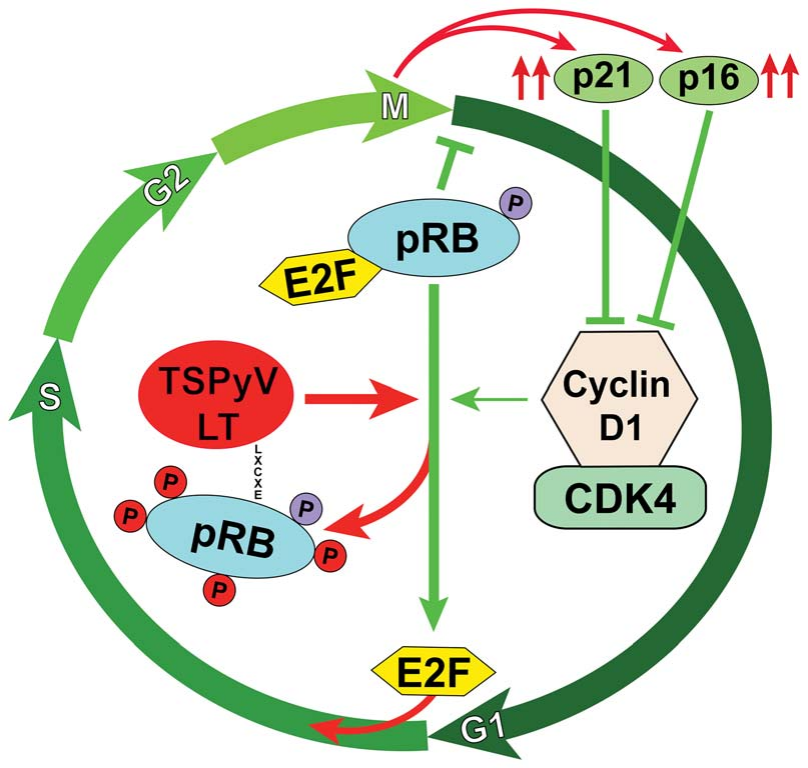
A hypothetical scenario of TSPyV LT-antigen interference in cell cycle regulation. An oversimplified cell-cycling scenario is shown that envisions the TSPyV LT-antigen involvement in regulation of pRB pathway activity. In a normal physiological condition, hypophosphorylated pRB is complexed with transcription factor E2F during early G1 (rest) phase of the cell cycle. When pRB is hyperphosphorylated at specific residues by Cyclin-dependent kinases (CDK) coupled to Cyclin-D1, E2F is released that activates expression of growth stimulatory genes needed for the cells to enter the S (DNA synthesis) phase. pRB phosphorylation is under tight regulation of p16*^ink4a^* and p21*^waf^*. Hypothetically, through its conserved LXCXE motif TSPyV LT-antigen interacts with pRB/E2F complex to dissociate these proteins via pRB hyperphosphorylation, resulting into S phase entry and subsequent increased expression of p16*^ink4a^* and p21*^waf^* as a negative cell cycling feedback (red arrows).

Retinoblastoma family proteins (i.e., pRB, p107 and p130) are important regulators of the G1- (rest) to S-phase (DNA synthesis) transition of cells during cell cycle progression. For instance, hypophosphorylated pRB inhibits function of the E2F transcription factor that regulates gene expression required for DNA synthesis. Hyperphosphorylation of pRB by complexes of Cyclin-D and cyclin-dependent kinases (CDKs) results into pRB–E2F complex dissociation and cell-cycle entry, which is reverted by several inhibitory proteins such as p16*^ink4a^* and p21*^waf^* [25]. Demonstration of phosphorylated pRB colocalized with Ki-67 indicates pRB-inactivation in the TS lesions and suggests progression into the S-phase (**Figure 5**). The observed increased expression of p16*^ink4a^* and p21*^waf^*, factors that normally inhibit Cyclin-D1/CDK activity, is explained as a negative feedback mechanism to inhibit cell cycle progression [25]. Unfortunately, because of lack of additional TS lesional samples/sections, we were unable to look into other cell cycle-regulatory pathways, for example involving p53, which can be revoked by polyomaviruses as well [12], [26]–[28].

The observed pattern of cell cycle deregulation and S-phase progression through hyperphosphorylation/inactivation of pRB is also seen in HPV16-induced cervical dysplasia and neoplasia [15]. In that case, phosphorylation of pRB is mediated by the E7 viral oncoprotein, comparable to the action of LT-antigen of SV40 [24]. Crucial to the inactivation of pRB is binding by LT-antigen and E7 through a conserved LXCXE motif found in these viral oncoproteins, and in TSPyV LT-antigen as well [6], [10]. Whether TSPyV LT-antigen interacts with pRB and hampers its function, for instance by hyperphosphorylation, requires experimental confirmation. Especially, since in our analyses technical limitations (shared origin of the antibody, Table 2) prevented discrimination between TSPyV LT-antigen and phosphorylated pRB, and therefore, the ability to demonstrate colocalization of both markers. Since the TS follicles were pRB-positive, it is unlikely that TSPyV LT-antigen promotes pRB degradation next to hyperphosphorylation, as is known for HPV16 (Supplementary Figure 1).

Taken together, our findings are compatible with a scenario of TSPyV LT-antigen-induced cell cycle progression through disruption of pRB-regulatory pathways, thereby creating a reservoir of proliferating IRS cells that enable viral DNA replication. Terminal differentiation of this large pool of IRS cells could explain the final accumulation of trichohyalin-positive cells and the formation of TS-characteristic spicules.

## Supporting Information

Figure S1 **Organotypic raft cultures used as staining controls.** H&E staining (**A1** and **B1**), trichohyalin staining (TCHH) (**A2** and **B2**) and Ki-67 staining (**A3** and **B3**) in organotypic raft cultures expressing empty vector (pLZRS) (Mock) or HPV16 oncogenes E6/E7 are shown in the upper group of figures. Note many suprabasal mitotic cells in B1 (**inset**). In the lower group of figures, staining for cell cycle regulatory proteins, p16*^ink4a^* (**C1** and **D1**), p21*^waf^* (**C2** and **D2**) and pRB (**C3** and **D3**) in Mock rafts and HPV16 rafts are shown. Some (secondary antibody) nonspecific staining of the cornified layer was present in all materials tested in this study. The dermoepidermal junction is indicated by dotted lines. Bar depicts 100 µm.
(TIF)
