# Supplementary figures and images for: Polyomavirus-Associated Trichodysplasia Spinulosa Involves Hyperproliferation, pRB Phosphorylation and Upregulation of p16 and p21
Source: PLoS One. 2014 Oct 7;9(10):e108947. doi: 10.1371/journal.pone.0108947 (PMC4188587; doi:10.1371/journal.pone.0108947)

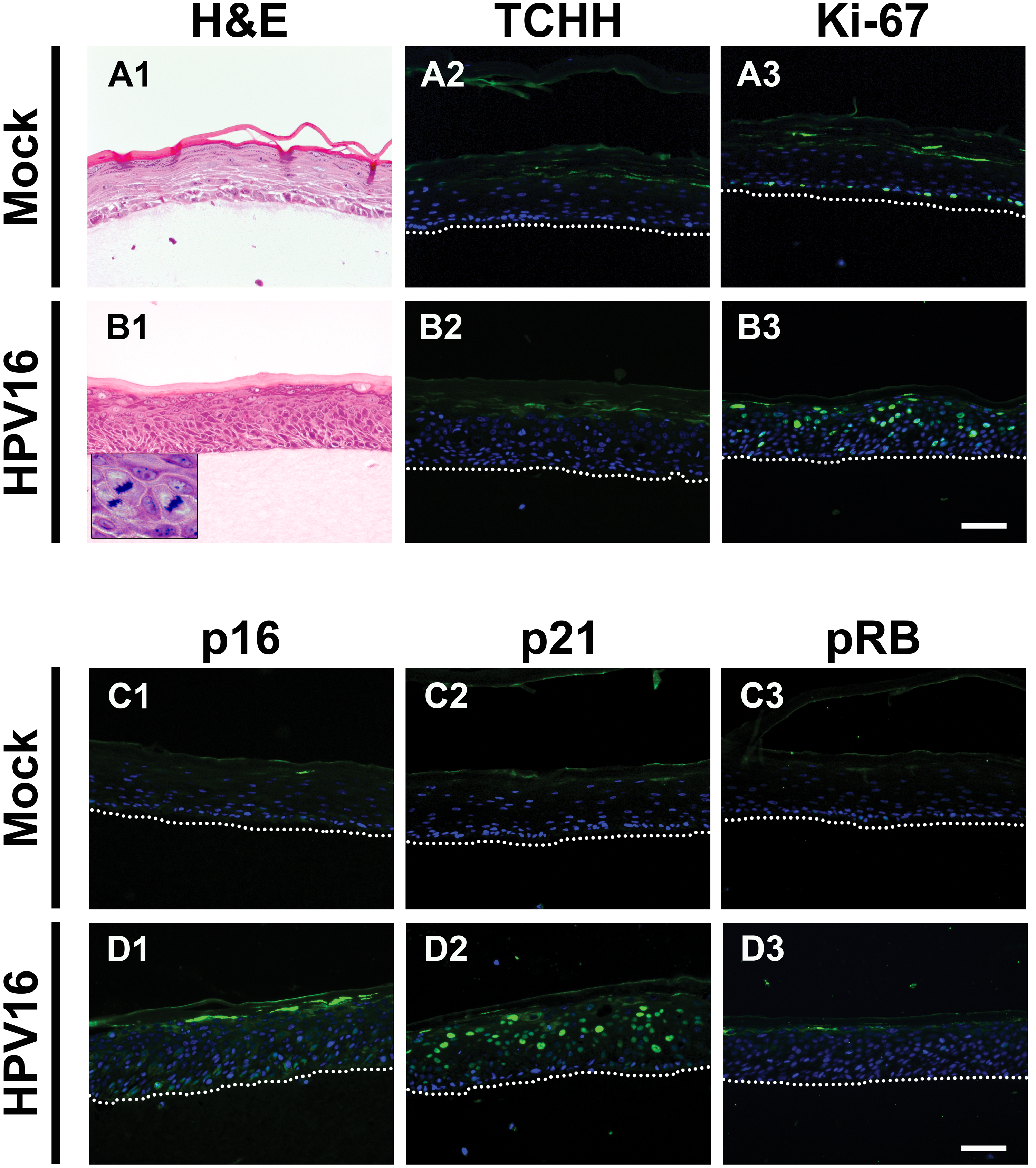

Supplement: Figure S1 — Organotypic raft cultures used as staining controls. H&E staining (A1 and B1), trichohyalin staining (TCHH) (A2 and B2) and Ki-67 staining (A3 and B3) in organotypic raft cultures expressing empty vector (pLZRS) (Mock) or HPV16 oncogenes E6/E7 are shown in the upper group of figures. Note many suprabasal mitotic cells in B1 (inset). In the lower group of figures, staining for cell cycle regulatory proteins, p16ink4a (C1 and D1), p21waf (C2 and D2) and pRB (C3 and D3) in Mock rafts and HPV16 rafts are shown. Some (secondary antibody) nonspecific staining of the cornified layer was present in all materials tested in this study. The dermoepidermal junction is indicated by dotted lines. Bar depicts 100 µm. (TIF) [file pone.0108947.s001.tif]
